# Tunable magnetization and damping of sputter-deposited, exchange coupled Py|Fe bilayers

**DOI:** 10.1038/s41598-017-05030-8

**Published:** 2017-07-07

**Authors:** Pavlo Omelchenko, Eric Arturo Montoya, Chris Coutts, Bret Heinrich, Erol Girt

**Affiliations:** Simon Fraser University, Department of Physics, Burnaby, BC V5A 1S6 Canada

## Abstract

We report on magnetic damping of exchange coupled, polycrystalline Py(Ni_80_Fe_20_)|Fe and Fe|Py bilayers, prepared by sputter-deposition on an amorphous 3 nm Ta seed layer. FMR measurements are performed on varying thicknesses of the individual Py and Fe layers while keeping the total bilayer structure thickness fixed. When Fe is grown directly on Ta, there is large magnetic inhomogeneity and damping. However, when a Py layer is deposited between Fe and Ta, both the magnetic inhomogeneity and damping significantly decrease even if Fe is covered by Ta. The intrinsic damping of the Ta|Py|Fe film can be further lowered by increasing the Fe to Py ratio. SQUID measurements show a linear increase in saturation magnetization with increasing ratio of Fe to Py. A combination of in-plane and out-of-plane X-ray diffraction measurements show that Py is textured along the 〈111〉 directions and Fe is textured along the 〈110〉, with Fe texture significantly improving if it is deposited on Ta|Py instead of Ta. By improving the texture of Fe by introducing a thin Py layer between Fe and Ta, one can grow Fe thin films with zero in-plane anisotropy, tunable magnetic moment, and low magnetic damping, approaching that of the best single crystal Fe.

## Introduction

Damping is one of the key parameters controlling magnetization dynamics and thus has been actively studied for decades^1–3^. Many studies focus on understanding the origins of magnetic damping in hopes of creating low magnetic damping materials. From the experimental research point of view, low damping implies higher measurement sensitivity for magnetization dynamics experiments, such as spin pumping^4–6^. For applications, such as spin torque magnetic random access memory (ST-MRAM); spin torque oscillators; and magnonics, low damping materials result in lower power consumption. Currently, the lowest known damping material is yttrium iron garnet (YIG)^7, 8^, with Gilbert damping values as low as 10^−5^. However, the manufacturing process of YIG imposes many constraints on the design of a research study or a device. Alternative low damping materials include single-crystal films^9^ or Heusler alloys^10^, but they suffer from the same drawbacks as YIG, requiring very specialized growth techniques, single-crystal substrates, and often high temperature annealing processes. Sputter deposition is one of the most popular and versatile growth techniques used in industry for the deposition of a large variety of metal and oxide materials. Magnetic films used in applications are often deposited on top of polycrystalline seed layers, which results in an increase in extrinsic damping of magnetic layers due to effects such as two-magnon scattering^11, 12^, sample inhomogeneity, or inplane nonlocal damping^13^. Recently, ultra-low intrinsic damping has been observed in 10 nm thick polycrystalline CoFe alloy films grown on top of a textured non-magnetic Cu seedlayer^14^. The metallic nature of the sample makes it attractive for applications involving the flow of charge current such as spin transfer torque in random access memory.

In this work we present an alternative metallic thin film magnetic structure with adjustable magnetic damping and magnetization, prepared by sputter-deposition. The structure is an exchange coupled bilayer of Permalloy (Py = Ni_80_Fe_20_) and Iron (Fe), where Py serves as a magnetic seedlayer that sets the texture for growth of Fe layer. Magnetic properties of the individual layers are well documented in scientific literature. The magnetic bilayer, Py|Fe has the traits of both layers adjustable by their relative thicknesses. We investigate the magnetic damping, magnetization, and crystal structure of Py|Fe, as well as Fe|Py, magnetic bilayers deposited on a Ta seed layer by means of ferromagnetic resonance (FMR), superconducting superconducting quantum interference device (SQUID), and X-ray diffraction (XRD). The results of our studies yield a sputter-deposited bilayer structure with an adjustable magnetization, from bulk Py to bulk Fe, which is important for devices manufacturing. Furthermore, the damping can be adjusted to values approaching that of perfect single crystal Fe damping^15^. Such a result brings the utility of the high magnetization and low damping of single crystal Fe, an important material in fundamental studies^16–18^, into sputter-deposited samples. This structure is fundamentally different to the CoFe alloy structure, mentioned above, since it has two individual magnetic layers and interfaces. This structure has already exploited in the design of a nonlocal damping study^19^ where it was necessary to have a magnetic layer, acting as a spin sink, with a Py interface but significantly improved magnetic dynamic properties from that of Py.

## Results

### Structural properties

**Figure 1:**
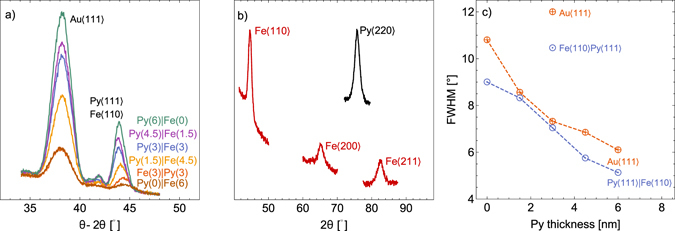
(**a**) Out-of-plane XRD *θ* − 2*θ* scan for Py|Fe and Fe|Py bilayers deposited on Ta and capped with Au. Layer thickness in nm are labeled in parenthesis. The peak at 38.1° is from 〈111〉 Au protective layer and the peak between 44.0–44.4° is from both 〈111〉 Py (bulk at 44.0°) and 〈110〉 Fe (bulk at 44.4°). (**b**) In-plane XRD at 0.5° grazing incidence angle of ${\rm{Ta}}(3)|{\bf{Py}}({\bf{1.5}})|{\bf{Fe}}({\bf{10.5}})|{\rm{Ta}}(3)$ (bottom peaks) and ${\rm{Ta}}(3)|{\bf{Py}}({\bf{12}})|{\rm{Ta}}(3)$ (top peak). (**c**) FWHM of XRD rocking curve of  Au and  Py|Fe peaks of ${\rm{Ta}}(3)|{\bf{Py}}({{\boldsymbol{d}}}_{{\rm{Py}}})|{\bf{Fe}}({\bf{6}}-{{\boldsymbol{d}}}_{{\rm{Py}}})|{\rm{Au}}(3.6)$, dashed lines are guide for the eye. The two outlier points correspond to the ${\rm{T}}{\rm{a}}(3)|{\bf{F}}{\bf{e}}({\bf{3}})|{\bf{P}}{\bf{y}}({\bf{3}})|{\rm{A}}{\rm{u}}(3.6)$ structure.

All samples were grown on a 3 nm Ta seed layer deposited on Si substrates. The main sample structure of interest is Si|Ta(3)|**Py**(***d***_Py_)**|Fe**(**6** − ***d***_Py_)|Au(3.6), where the numbers in parenthesis refer to the layer thicknesses in nm. The Py layer thickness is given as *d*_Py_, and the total thickness of the magnetic bilayer is kept constant at 6 nm. For comparison, structures with the Py and Fe stack order switched, Fe|Py, are also investigated. Out-of-plane, X-ray diffraction (XRD) measurements were performed on Si|Ta(3)|**Py**(***d***_Py_)|**Fe**(**6** − ***d***_Py_)|Au(3.6) films structures for *d*_Py_ = 0, 1.5, 3.0, 4.5, 6.0 nm, see Fig. 1a). In-plane XRD of Si|Ta(3)|**Py**(**1**.**5**)|**Fe**(**10**.**5**)|Ta(3) and Si|Ta(3)|**Py**(**12**)|Ta(3) are displayed in Fig. 1b). The magnetic layer thickness for in-plane XRD were increased to improve instrument sensitivity. The in-plane XRD samples were capped with an amorphous Ta(3)^19^ instead Au(3.6) to avoid interference of the polycrystalline Au in XRD data. Both out-of-plane and in-plane measurements show that Py grows along the 〈111〉 directions when deposited on Ta, the brackets represent a family of directions. In-plane XRD measurements show that Fe grown on Py is textured along the 〈110〉 crystallographic axes. Full width at half maximum (FWHM) of XRD rocking curves of the Au〈111〉 and Py〈111〉|Fe〈110〉 peaks for the ${\rm{Ta}}(3)|{\bf{Py}}({{\boldsymbol{d}}}_{{\rm{Py}}})|{\bf{Fe}}({\bf{6}}-{{\boldsymbol{d}}}_{{\rm{Py}}})|{\rm{Au}}(3.6)$ and ${\rm{Ta}}(3)|{\bf{Fe}}({\bf{3}})|{\bf{Py}}({\bf{3}})|{\rm{Au}}(3.6)$ samples are plotted as a function of Py thickness in Fig. 1c). The figure shows an increase in the texture of both Au and Py|Fe layers with increasing thickness of Py. If Fe is grown directly on top of Ta the film texture deteriorates. The increase in texture results in the increase of peak intensity in the out-of-plane XRD scans in Fig. 1a). The texture of the Au capping layer is also improved as the result of the improved texture of Py|Fe film. For the rest of the paper the ${\rm{Si}}|{\rm{Ta}}(3)|{\bf{Py}}({{\boldsymbol{d}}}_{{\rm{Py}}})|{\bf{Fe}}({\bf{6}}-{{\boldsymbol{d}}}_{{\rm{Py}}})|{\rm{Au}}(3.6)$ and ${\rm{S}}{\rm{i}}|{\rm{T}}{\rm{a}}(3)|{\bf{F}}{\bf{e}}({\bf{3}})|{\bf{P}}{\bf{y}}({\bf{3}})|{\rm{A}}{\rm{u}}(3.6)$ structures are abbreviated as ${\bf{Py}}({{\boldsymbol{d}}}_{{\rm{Py}}})|{\bf{Fe}}({\bf{6}}-{{\boldsymbol{d}}}_{{\rm{Py}}})$ and ${\bf{Fe}}({\bf{3}})|{\bf{Py}}({\bf{3}})$ respectively, it should be assumed a Ta(3) seed layer and a Au(3.6) capping layer unless otherwise stated.

### Magnetic properties

#### SQUID magnetometry

**Figure 2:**
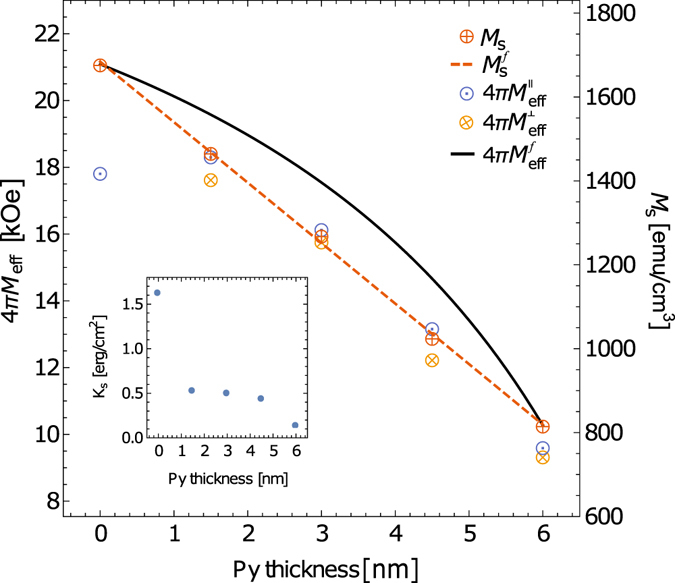
The saturation magnetization *M*_s_ as determined from SQUID measurements (right axis) and effective saturation induction 4*πM*_eff_ as determined by FMR (left axis) for the ${\bf{Py}}({{\boldsymbol{d}}}_{{\rm{Py}}})|{\bf{Fe}}({\bf{6}}-{{\boldsymbol{d}}}_{{\rm{Py}}})$ structure. Left axis is scaled by 4*π* to right axis for direct comparison. The dashed line represents a linear fit to the *M*_s_ data using eq. (1) and the solid line is a plot of eq. (2) using ${M}_{{\rm{s}}}^{{\rm{Py}}}=817\,{\rm{emu}}/{{\rm{cm}}}^{3}$ and ${M}_{{\rm{s}}}^{{\rm{Fe}}}=1678\,{\rm{emu}}/{{\rm{cm}}}^{3}$. The interface anisotropy as extracted from eq. (9) is shown in the inset.

The saturation magnetization, *M*_s_, of ${\bf{Py}}({{\boldsymbol{d}}}_{{\rm{Py}}})|{\bf{Fe}}({\bf{6}}-{{\boldsymbol{d}}}_{{\rm{Py}}})$ and ${\bf{Fe}}({\bf{3}})|{\bf{Py}}({\bf{3}})$ films was determined by means of SQUID magnetometry and plotted in Fig. 2. A linear decrease in *M*_s_ is observed with an increasing ratio of Py to Fe. One can calculate the expected trend of *M*_s_ as a function of Py to Fe ratio by weighting the individual magnetizations by the corresponding thicknesses of each material,

1 $${M}_{{\rm{s}}}^{f}=\frac{{M}_{{\rm{s}}}^{{\rm{Py}}}{d}_{{\rm{Py}}}}{{d}_{{\rm{Py}}}+{d}_{{\rm{Fe}}}}+\frac{{M}_{{\rm{s}}}^{{\rm{Fe}}}{d}_{{\rm{Fe}}}}{{d}_{{\rm{Py}}}+{d}_{{\rm{Fe}}}}.$$

where ${M}_{{\rm{s}}}^{{\rm{Py}}}$ and ${M}_{{\rm{s}}}^{{\rm{Fe}}}$ are the saturation magnetization of Py and Fe, respectively, and *d*_Py_ and *d*_Fe_ are the thicknesses of Py and Fe layers, respectively. It turns out that the SQUID measurements overlap with the expected average saturation of magnetization obtained using bulk values of the **Py** (${M}_{{\rm{s}}}^{{\rm{Py}}}=817\pm 7\,{\rm{emu}}/{{\rm{cm}}}^{3}$) and **Fe** (${M}_{{\rm{s}}}^{{\rm{Fe}}}=1678\pm 7\,{\rm{emu}}/{{\rm{cm}}}^{3}$). This indicates high quality of the sputtered films; defects that would reduce *M*_s_, such a film interface oxidation, are not present in the structures.

The effective saturation induction, 4*πM*_eff_, of a bilayer system assuming no anisotropy can be determined using^16^,

2 $$4\pi {M}_{{\rm{eff}}}^{f}=4\pi \frac{{({M}_{{\rm{s}}}^{{\rm{Py}}})}^{2}{d}_{{\rm{Py}}}}{{M}_{{\rm{s}}}^{{\rm{Py}}}{d}_{{\rm{Py}}}+{M}_{{\rm{s}}}^{{\rm{Fe}}}{d}_{{\rm{Fe}}}}+4\pi \frac{{({M}_{{\rm{s}}}^{{\rm{Fe}}})}^{2}{d}_{{\rm{Fe}}}}{{M}_{{\rm{s}}}^{{\rm{Py}}}{d}_{{\rm{Py}}}+{M}_{{\rm{s}}}^{{\rm{Fe}}}{d}_{{\rm{Fe}}}}.$$

The plot of eq. (2) using ${M}_{{\rm{s}}}^{{\rm{Py}}}$ and ${M}_{{\rm{s}}}^{{\rm{Fe}}}$ from SQUID are shown as black solid line on Fig. 2.

#### Ferromagnetic Resonance

Magnetization dynamics of a thin magnetic film, undergoing ferromagnetic resonance (FMR), are well described by the Landau-Lifshitz-Gilbert equation,

3 $$\frac{\partial {\boldsymbol{M}}}{\partial t}=-\gamma [{\boldsymbol{M}}\times {{\boldsymbol{H}}}_{{\rm{eff}}}]+\alpha [{\boldsymbol{M}}\times \frac{\partial {\boldsymbol{n}}}{\partial t}],$$

where ***M*** is the instantaneous magnetization vector with magnitude *M*_s_, ***n*** is the unit vector parallel to ***M***, ***H***_eff_ is the sum of internal and external *H*-fields, and *α* is the dimensionless Gilbert damping parameter.

The FMR resonance condition for a textured, thin film, with zero in-plane anisotropy, in the in-plane geometry, is given by

4 $${(\frac{\omega }{\gamma })}^{2}=({H}_{{\rm{FMR}}})\,({H}_{{\rm{FMR}}}+4\pi {M}_{{\rm{eff}}}^{\parallel }),$$

where $\omega$ is the microwave frequency, $\gamma =g{\mu }_{B}/\hslash$, *g* is the Landé *g*-factor, *μ*_*B*_ is the Bohr magneton, $\hslash$ is the reduced Planck constant, *H*_FMR_ is the resonance field, $4\pi {M}_{{\rm{eff}}}^{\parallel }=4\pi {M}_{{\rm{s}}}-2{K}_{{\rm{u}}}^{\perp }/{M}_{{\rm{s}}}$ and ${K}_{{\rm{u}}}^{\perp }$ is the perpendicular-to-plane uniaxial anisotropy. Due to the polycrystalline nature of the samples, the in-plane magnetocrystalline anisotropy is averaged out and therefore not observed in the in-plane FMR measurements.

For perpendicular-to-plane FMR, resonance condition is given by

5 $$\frac{\omega }{\gamma }={H}_{{\rm{FMR}}}+4\pi {M}_{{\rm{eff}}}^{\perp }.$$

For samples textured in out-of-plane geometry, the magnetocrystalline anisotropy is no longer average out and can play a significant role, $4\pi {M}_{{\rm{eff}}}^{\perp }=4\pi {M}_{{\rm{s}}}-2{K}_{{\rm{u}}}^{\perp }/{M}_{{\rm{s}}}-{K}_{4}/{M}_{{\rm{s}}}$.

The FMR linewidth is well described by Gilbert-like damping,

6 $${\rm{\Delta }}H(\omega )=\alpha \frac{\omega }{\gamma }+{\rm{\Delta }}H\mathrm{(0}),$$

where Δ*H*(0) is the zero-frequency line broadening due to magnetic inhomogeneity^1, 16, 20^ and the slope determines the effective damping parameter *α*.

**Figure 3:**
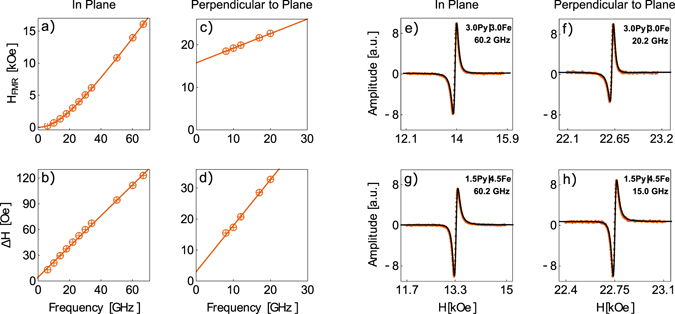
Examples of FMR data for in-plane and perpendicular-to-plane measurements of **Py**(**3**)|**Fe**(**3**) sample. The resonance fields, *H*_FMR_, and the resonance peak broadening, Δ*H*, as a function of frequency for (**a**,**b**), in-plane, and (**c**,**d**), perpendicular-to-plane external field configurations. FMR spectra of (**e**) **Py**(**3**)|**Fe**(**3**) in-plane at 20.2 GHz, (**f**) **Py**(**3**)|**Fe**(**3**) perpendicular-to-plane at 60.2 GHz, (**g**) **Py**(**1**.**5**)|**Fe**(**4**.**5**) in-plane at 15 GHz, and (**h**) **Py**(**1**.**5**)|**Fe**(**4**.**5**) perpendicular-to-plane at 60.2 GHz. The solid line is a perfect fit to the signal by assuming an admixture of the out-of-phase and in-phase components of RF susceptibility^21^, this is a common procedure for measurements on transmission line and terminated waveguides.

In-plane and perpendicular-to-plane FMR measurements where carried out on a coplanar waveguide for a frequency range of 6–36 GHz. In-plane FMR was also performed on a terminated rectangular waveguide for a frequency range of 50–67 GHz, see Fig. 3(a), as detailed in ref. 21. The resonance field measurements were interpreted by using eq. (4). Fitting of the in-plane and perpendicular-to-plane FMR data was performed using eqs (4) and (5). During the fitting the *g*–factor was constrained between *g* = 2.09 − 2.10 in order to be consistent with reported literature values: *g*_Fe_ = 2.09^9, 15^ and *g*_Py_ = 2.10^22–24^.

## Discussion

**Figure 4:**
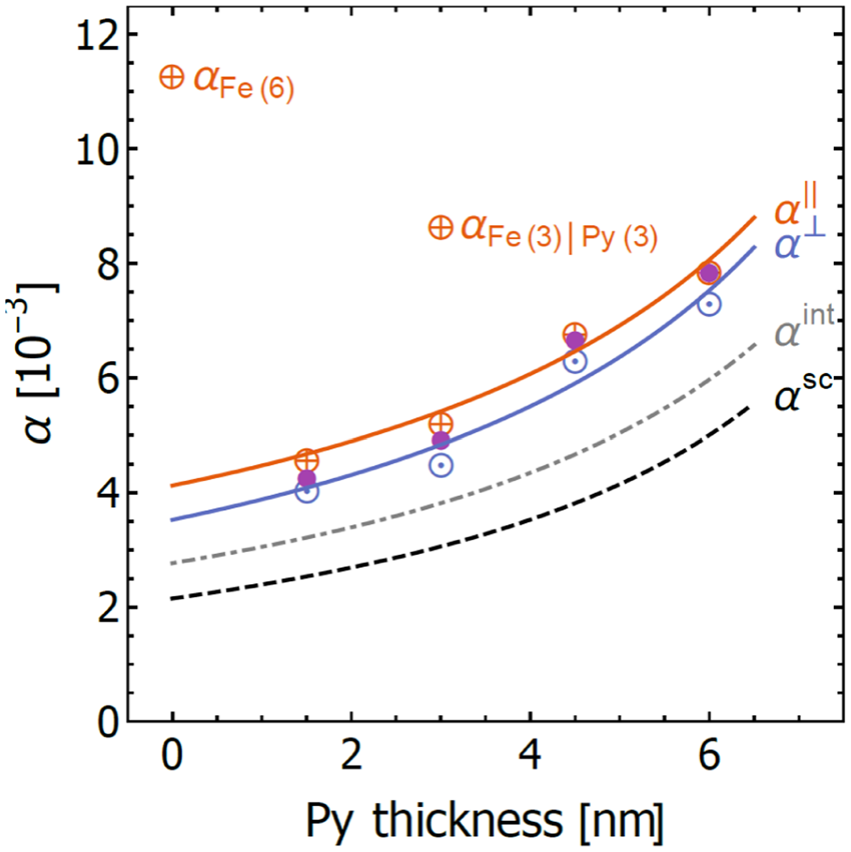
Gilbert damping as function of Py thickness (*d*_Py_ = 0, 1.5, 3.0, 4.5, and 6.0 nm) in magnetic bilayer ${\bf{Py}}({{\boldsymbol{d}}}_{{\rm{Py}}})|{\bf{Fe}}({\bf{6}}-{{\boldsymbol{d}}}_{{\rm{Py}}})$. Data is shown for the in-plane all frequencies (), in-plane high frequencies 50–67 GHz (), and perpendicular-to-plane () FMR configurations. The solid lines, ${\alpha }^{\perp }$ and ${\alpha }^{\parallel }$, are fits to the data using eq. (7). The two outlier points show very large enhancement in damping for samples where Fe was deposited before Py. *α* ^int^ is the fit of the perpendicular-to-plane damping using eq. (7), with the contribution on due to spin pumping in Ta subtracted off. The fitting parameters are ${\alpha }_{{\rm{Py}}}^{{\rm{int}}}={\alpha }_{{\rm{Py}}}^{\perp }-{\alpha }_{{\rm{Py}}}^{{\rm{sp}}}=\mathrm{6.0(4)}\times {10}^{-3}$ and ${\alpha }_{{\rm{Fe}}}^{{\rm{int}}}=\mathrm{2.8(4)}\times {10}^{-3}$. *α* ^*sc*^ is an estimate of the damping for bulk single-crystal structure of Py|Fe from eq. (7).

The main result of this work is displayed in Fig. 4. In-plane FMR measurements of Fe sputtered directly on Ta yields a high value for both Δ*H*(0) = 96 Oe and ${\alpha }_{{\rm{Fe}}(6)}^{\parallel }=11.5\times {10}^{-3}$. This can be improved if Py is deposed on top of Fe as in the **Fe**(**3**)|**Py**(**3**) sample with Δ*H*(0) = 20 Oe and ${\alpha }_{{\rm{Fe}}(3)|{\rm{Py}}(3)}^{\parallel }=8.7\times {10}^{-3}$. However, with the insertion of a thin layer of Py before the growth of Fe, the magnetic dynamic properties improve dramatically. For the **Py**(**1**.**5**)|**Fe**(**4**.**5**) structure, the magnetic damping is reduced by a factor of 3 to ${\alpha }_{{\rm{Py}}(1.5)|{\rm{Fe}}(4.5)}^{\parallel }=4.6\times {10}^{-3}$ and Δ*H*(0) = 7 Oe. This is almost two times lower than the damping of the pure Py sample, **Py**(**6**), ${\alpha }_{{\rm{Py}}(6)}^{\parallel }=7.9\times {10}^{-3}$. The damping measured in our **Py**(**6**) sample is the same as that reported for bulk Py^25, 26^. The large reduction in Δ*H*(0) with the insertion of a thin Py layer can be attributed to exchange narrowing^27^. A distribution of grains, and their orientations, will lead to a distribution of resonant fields leading to a large magnetic inhomogeneity. As the grains become small enough, and more textured, the exchange field will decrease the variations of internal fields by the exchange narrowing effect.

Perpendicular-to-plane FMR measurements, solid blue line in Fig. 4, yield a damping consistently lower than the in-plane, solid red line Fig. 4, by ${\rm{\Delta }}\alpha ={\alpha }^{\parallel }-{\alpha }^{\perp }=0.7\times {10}^{-3}$. One possibility is that the discrepancy is due to two-magnon scattering in the in-plane FMR^11^. The origin of two-magnon scattering is due the presence of spin-wave modes degenerate with the FMR mode. In the perpendicular-to-plane orientation there are no magnons degenerate to FMR modes and therefore two-magnon scattering does not contribute to the measured damping^16^. Evidence of two-magnon contribution is present in the dependence of in-plane Δ*H* in Fig. 3b), which show a slight deviation from linear behavior at low frequencies, <15 GHz. The lowest total measured damping is ${\alpha }^{\perp }=4.1\times {10}^{-3}$ for the **Py**(**1**.**5**)|**Fe**(**4**.**5**) structure. Since the two-magnon scattering contribution saturates at higher frequencies, we extracted the in-plane damping for the high frequency (50–67 GHz) and low frequency (6–14 GHz) measurements separately using eq. (6). The low frequency fits yield larger damping, ${\alpha }_{LowFreq.}^{\parallel }=5.7\times {10}^{-3}$ and Δ*H* = 1.4 Oe for the **Py**(**1**.**5**)|**Fe**(**4**.**5**) sample. The high frequency data yield lower damping, ${\alpha }_{HighFreq.}^{\parallel }=4.3\times {10}^{-3}$, and Δ*H* = 12 Oe for **Py**(**1**.**5**)|**Fe**(**4**.**5**). For this sample the ${\alpha }_{HighFreq.}^{\parallel }$ and ${\alpha }^{\perp }$ are very similar, suggesting that the discrepancy is mostly due to two-magnon scattering. However, for the thicker Py samples the difference between ${\alpha }_{HighFreq.}^{\parallel }$ and ${\alpha }^{\perp }$ increases, see Fig. 4. Infact, for the **Py**(**6**) sample there is no evidence for two-magnon behavior in the measured frequency range, ${\alpha }_{HighFreq.}^{\parallel }={\alpha }_{LowFreq.}^{\parallel }$. The discrepancy between ${\alpha }^{\perp }$ and ${\alpha }^{\parallel }$ for the thicker Py samples could be because two-magnon scattering saturates at a much higher frequency. Alternatively, it could be due to the difference in intrinsic damping of the two orientations, not uncommon for thin film structures.

The damping measured by FMR is the total damping of the entire bilayer structure, averaged by the relative magnetic moment of each layer. Given that the damping of Py is relatively high, it must mean that the damping of the Fe layer deposited on top of Py is quite low. A quantitative analysis of damping can be done by assuming strong coupling between Py and Fe^16^,

7 $${\alpha }_{{\rm{Py}}|{\rm{Fe}}}={\alpha }_{{\rm{Py}}}\frac{{d}_{{\rm{Py}}}{M}_{{\rm{s}}}^{{\rm{Py}}}}{{d}_{{\rm{Py}}}{M}_{{\rm{s}}}^{{\rm{Py}}}+{d}_{{\rm{Fe}}}{M}_{{\rm{s}}}^{{\rm{Fe}}}}+{\alpha }_{{\rm{Fe}}}\frac{{d}_{{\rm{Fe}}}{M}_{{\rm{s}}}^{{\rm{Fe}}}}{{d}_{{\rm{Py}}}{M}_{{\rm{s}}}^{{\rm{Py}}}+{d}_{{\rm{Fe}}}{M}_{{\rm{s}}}^{{\rm{Fe}}}}.$$

Results of fitting the in-plane damping data using the above equation are: ${\alpha }_{{\rm{Py}}}^{\parallel }=\mathrm{8.1(3)}\times {10}^{-3}$ and ${\alpha }_{{\rm{Fe}}}^{\parallel }=\mathrm{4.1(3)}\times {10}^{-3}$, see red solid line in Fig. 4. Fitting the perpendicular-to-plane damping data leads to: ${\alpha }_{{\rm{Py}}}^{\perp }=\mathrm{7.5(4)}\times {10}^{-3}$ and ${\alpha }_{{\rm{Fe}}}^{\perp }=\mathrm{3.5(4)}\times {10}^{-3}$, see blue red line in Fig. 4.

Finally, it is interesting to determine and compare the intrinsic damping of the polycrystalline structure to the expected values from an equivalent bilayer of single crystal materials. The total damping is the sum of the intrinsic and extrinsic dampings, where the extrinsic arises from the spin pumping of Py|Fe into the seed Ta layer, *α* = *α* ^int^ + *α* ^sp^. The enhancement in damping due to spin pumping is give by ref. 28

8 $${\alpha }^{{\rm{sp}}}=\frac{g{\mu }_{B}}{4\pi {M}_{s}}\frac{{\tilde{g}}_{\uparrow \downarrow }}{{d}_{FM}}[1+\frac{{\tilde{g}}_{\uparrow \downarrow }\rho {e}^{2}{\lambda }_{{\rm{sd}}}}{2\pi \hslash \,\tanh \,({d}_{Ta}/{\lambda }_{{\rm{sd}}})}]$$

where, *ρ* is the resistivity of Ta metal, *e* is the fundamental charge, *d*_*FM*_ is the thickness of the ferromagnet (*d*_*Py*_ + *d*_*Fe*_), ${\tilde{g}}_{\uparrow \downarrow }$ is the renormalized spin mixing conductance which determines the efficiency of spin pumping and depends on the Ta|Py interface, *M*_s_ is the average saturation magnetization of the entire ferromagnet given by eq. (1), and *λ*_sd_ is the diffusion length of Ta. Using the parameters from Montoya *et al*.^19^ for *λ*_sd_ = 1.0 nm, *ρ* = 2.75 × 10^−16^ s, and ${\tilde{g}}_{\uparrow \downarrow }=1.5\times {10}^{15}\,{{\rm{cm}}}^{-2}$ as well as *M*_s_ from SQUID measurements in Fig. 2, one can determine the spin pumping into Ta for any ${\bf{Py}}({{\boldsymbol{d}}}_{{\rm{Py}}})|{\bf{Fe}}({\bf{6}}-{{\boldsymbol{d}}}_{{\rm{Py}}})$ structure. The intrinsic damping, *α* ^int^, is obtained by subtracting the spin pumping contribution, *α* ^sp^, from perpendicular-to-plane damping obtained from fit using eq. (7), the dashed gray line in Fig. 4. For **Py**(**1**.**5**)|**Fe**(**4**.**5**), the perpendicular-to-plane damping, ${\alpha }^{\perp }$, without spin pumping, is ${\alpha }^{{\rm{int}}}=\alpha -{\alpha }^{{\rm{sp}}}=3.0\times {10}^{-3}$, approaching the damping for perfect single crystal Fe^15^, ${\alpha }_{{\rm{Fe}}}\sim 2.1\times {10}^{-3}$. We also estimate the intrinsic damping for an equivalent single crystal Py|Fe structure, *α* ^sc^, by using bulk single crystal damping parameters of Py (${\alpha }_{{\rm{Py}}}^{{\rm{sc}}}=5\times {10}^{-3}$)^29^ and Fe (${\alpha }_{{\rm{Fe}}}^{{\rm{sc}}}=2.1\times {10}^{-3}$)^15^ in eq. (7) assuming *α* ^sp^ = 0, see black bottom dashed line in Fig. 4.

The anisotropy of the ${\bf{Py}}({{\boldsymbol{d}}}_{{\rm{Py}}})|{\bf{Fe}}({\bf{6}}-{{\boldsymbol{d}}}_{{\rm{Py}}})$ and **Fe**(**3**)|**Py**(**3**) can be determined by comparing the measured 4*πM*_eff_ to the expected values calculated from eq. (2), black solid line in Fig. 2. All the measured values are lower than what is expected from an anisotropy free structures, indicating a positive uniaxial anisotropy perpendicular to the film surface. The origin of the anisotropy is from multiple sources: surface roughness, interface anisotropy and magnetocrystalline anisotropy. The measured in-plane effective saturation $4\pi {M}_{{\rm{eff}}}^{\perp }$ is lower than $4\pi {M}_{{\rm{eff}}}^{\parallel }$ for all samples due to the magnetocrystalline anisotropy, which is averaged out for the in-plane geometry. Therefore the in-plane measurements can be used to extract information about the interface anisotropies, see inset in Fig. 2, by using,

9 $${K}_{s}=\frac{({d}_{{\rm{Py}}}{M}_{{\rm{s}}}^{{\rm{Py}}}+{d}_{{\rm{Fe}}}{M}_{{\rm{s}}}^{{\rm{Fe}}})}{2}\mathrm{(4}\pi {M}_{{\rm{eff}}}^{f}-4\pi {M}_{{\rm{eff}}}^{\parallel })$$

As expected, the interface anisotropy for the **Py**(**6**) sample is small. For the **Py**(**4**.**5**)|**Fe**(**1**.**5**), **Py**(**3**)|**Fe**(**3**) and **Py**(**1**.**5**)|**Fe**(**4**.**5**) samples the anisotropy increases to *K*_*s*_ ~ 0.5 erg/cm^2^, similar in value to the reported interface anisotropy of 〈110〉Fe|Au^30^. For the **Fe**(**6**) sample, the interface anisotropy jumps to *K*_*s*_ ~ 1.6 erg/cm^2^ due to the presence of both Ta|Fe and Fe|Au interfaces.

To test if the large increase in *α* of the **Fe**(**6**) vs. the **Py**(**1**.**5**)|**Fe**(**4**.**5**) is due to spin-orbit coupling at the interface of Ta|Fe, we deposited an additional sample, Si|Ta|**Py**(**1**.**5**)|**Fe**(**4**.**5**)|Ta(3), where we covered the Fe layer with Ta instead of Au. In this case the Fe shares interface with a Ta layer similar as in the case of **Fe**(**6**)|Au(3.6). We observe that for the ${\rm{Si}}|{\rm{Ta}}|{\bf{Py}}({\bf{1.5}})|{\bf{Fe}}({\bf{4.5}})|{\rm{Ta}}(3)$ sample the 4*πM*_eff_ is equal to that of ${\rm{Si}}|{\rm{Ta}}|{\bf{Py}}({\bf{1.5}})|{\bf{Fe}}({\bf{4.5}})|{\rm{Au}}(3.6)$. A small increase of *α* in ${\rm{Si}}|{\rm{Ta}}|{\bf{Py}}({\bf{1.5}})|{\bf{Fe}}({\bf{4.5}})|{\rm{Ta}}(3)$ in comparison ${\rm{Si}}|{\rm{Ta}}|{\bf{Py}}({\bf{1.5}})|{\bf{Fe}}({\bf{4.5}})|{\rm{Au}}(3.6)$ can be attributed to additional spin pumping at second Fe|Ta interface. It would suggest that spin-orbit coupling at the Ta|Fe interface plays no role increasing damping, however, one needs to be careful in interpreting this result. As can be observed from XRD data, Fig. 1a,c), the Fe deposited on amorphous Ta^19^ is poorly textured. In this case where Fe initially grows on Ta, the Fe layer may have grains oriented in different directions and after some thickness Fe grains that do not grow along the 〈110〉 crystal orientation get annihilated. As a result the two interfaces, Ta|Fe and Fe|Ta, are different. The Ta|Fe interface may be an ensemble of the different orientations of Fe grains on Ta such as: Ta|Fe〈110〉 and Ta|Fe〈100〉, while Fe|Ta almost entirely consists of Fe〈110〉|Ta grains. Peng *et al*.^31^ have shown that there is a large perpendicular interface anisotropy at Ta|FeCo〈100〉 interface. Since the Ta|Fe interface is a combination of Fe grains with different crystallographic orientation, a large difference in the interface anisotropy of the Ta|Fe〈100〉 and Ta|Fe〈110〉 would result in magnetic homogeneity throughout the Ta|Fe interface. This is consistent with the observed large zero frequency offset for the sample. The large inhomogeneity, in turn, will lead to an enhancement in damping due to non-uniform magnetization precession^13, 32, 33^. Additionally, each orientation may have different damping, as was calculated for Co|Pd system^34^. Therefore, we conclude that the large damping in the and structures is the result of variation of the Ta|Fe interface anisotropy caused by initial, poorly-textured growth of Fe on Ta that led to variation of the Ta|Fe interface anisotropy.

We have shown that sputter-deposited Fe films show much improved damping characteristics when they are grown on top of a thin Py insertion layer instead of directly on the Ta seed layer. Furthermore, by varying the Fe to Py layer thickness ratio one can tune magnetization from about 817 to 1467 emu/cm^3^ while keeping the magnetic damping below 0.08. For large Fe to Py layer thickness ratio the damping of Py|Fe approaches that of single crystal Fe films. The large damping in Fe films grown directly on top of Ta is result of the variation of the Ta|Fe interface anisotropy induced by a poor texture of Fe on top of Ta. Varying the relative thicknesses of Py and Fe in the Py|Fe bilayer allows one to tune *M*_s_ and *α* following simple models. Such layer can be used to elucidate nonlocal damping studies by using both spin pumping^35, 36^ and spin sink^5, 19, 37–39^ effects to investigate spin transport in multilayer systems, as the tunability of *M*_s_ can make the spin pump and spin sink have much different resonance fields. Using the spin sink effect has advantages similar to using the inverse spin Hall effect to detect spin pumping signals^40, 41^ as it is also purely nonlocal, with the advantage of being free of charge currents. Using such a exchange-coupled ferromagnetic layer as spin sink in a multilayer system with two effective ferromagnetic layers, one could use spin pump and spin sink effects to separate contributions to damping from spin pumping and recently proposed damping mechanisms such as spin memory loss^42^ and proximity effects^43, 44^.

## Methods

### Thin film deposition

Room temperature RF magnetron sputtering was used to deposit all film layers on oxidized Si wafers. sputter-deposition was performed at an argon pressure of 1.5 × 10^−3^ torr with a base pressure below 5 × 10^−8^ torr. Before deposition, the substrates were cleaned with acetone and isopropyl alcohol under ultrasonic conditions at a temperature of 333 K. In RCA cleaning, Si substrates are soaked for approximately 15 min in a solution of deionized (DI) water (60 mL), NH_4_OH (12 mL), and H_2_O_2_ (8 mL), that is kept at a temperature range of 343 ± 5 K. After the cleaning, the Si substrates are rinsed with DI water. The Ta(3) was used as a seed layer for Py(Ni_80_Fe_2_), allowing for 〈111〉 growth orientations. The (3.6)Au served as a protective layer to prevent oxidation of the structure. The layer thicknesses were determined from growth rate calibrated by X-ray reflectivity (XRR) measurements.

### X-Ray measurements

In-plane and out-of-plane XRD measurements were performed by means of a high resolution X-ray diffractometer (HRXRD) using Cu K_*α*_ radiation. The distances between lattice planes parallel and perpendicular to the film surfaces were measured with in-plane and out-of-plane XRD measurements, respectively. In both cases, we used Cu K_*α*_ radiation source. For the in-plane XRD measurements, the incident and scattered beams are nearly parallel to sample surface. This allows the measurements of spacing between lattice planes nearly perpendicular to the sample surface. In-plane XRD measurements were performed at a grazing angle of 0.5°. Out-of-plane XRD measurements were performed with the scattering wave vector normal to the film surface.

### Superconducting quantum interference device (SQUID) measurement

In-plane hysteresis curves were measured at 300 K to determine the magnetic moment of the samples. The samples were diced and the area measured by some means. With the ferromagnetic film thicknesses determined from XRR calibration, the ferromagnetic volume was calculated. The saturation magnetization *M*_s_ for the samples is then determined by normalizing the measured moment to the total volume of ferromagnetic material (Py + Fe) in the sample.

### Ferromagnetic resonance measurements

In-plane and perpendicular-to-plane FMR transmission measurements where carried out on a coplanar waveguide for a frequency range of 6–36 GHz. Additionally, in-plane FMR reflection measurements were also in terminated rectangular waveguide for a frequency range of 50–67 GHz. All FMR measurements were performed in a large nuclear magnetic resonance (NMR) magnet with field modulation coils in the Helmholtz configuration to ensure homogeneity of field. The microwave source was a broadband microwave generator and detection was by means of detector diodes, as detailed in refs^21^.
